# Changes in Protein O-GlcNAcylation During Mouse Epididymal Sperm Maturation

**DOI:** 10.3389/fcell.2018.00060

**Published:** 2018-06-11

**Authors:** Darya A. Tourzani, Bidur Paudel, Patricia V. Miranda, Pablo E. Visconti, María G. Gervasi

**Affiliations:** ^1^Department of Veterinary and Animal Sciences, Integrated Sciences Building, University of Massachusetts, Amherst, MA, United States; ^2^Instituto de Agrobiotecnología Rosario S.A. (INDEAR), Rosario, Argentina

**Keywords:** sperm maturation, O-GlcNAcylation, O-GlcNAc transferase, spermatozoa, epididymis

## Abstract

After leaving the testis, sperm undergo two sequential maturational processes before acquiring fertilizing capacity: sperm maturation in the male epididymis, and sperm capacitation in the female reproductive tract. During their transit through the epididymis, sperm experience several maturational changes; the acquisition of motility is one of them. The molecular basis of the regulation of this process is still not fully understood. Sperm are both transcriptionally and translationally silent, therefore post-translational modifications are essential to regulate their function. The post-translational modification by the addition of O-linked β-N-acetylglucosamine (O-GlcNAc) can act as a counterpart of phosphorylation in different cellular processes. Therefore, our work was aimed to characterize the O-GlcNAcylation system in the male reproductive tract and the occurrence of this phenomenon during sperm maturation. Our results indicate that O-GlcNAc transferase (OGT), the enzyme responsible for O-GlcNAcylation, is present in the testis, epididymis and immature caput sperm. Its presence is significantly reduced in mature cauda sperm. Consistently, caput sperm display high levels of O-GlcNAcylation when compared to mature cauda sperm, where it is mostly absent. Our results indicate that the modulation of O-GlcNAcylation takes place during sperm maturation and suggest a role for this post-translational modification in this process.

## Introduction

Mammalian fertilization is a complex process in which a spermatozoon fuses with an MII-arrested oocyte to form a new individual (Yanagimachi, 1994). The dynamic interactions that occur during fertilization depend on the proper function of gametes. Sperm are produced in the testis and released into the epididymis, where they undergo a post-testicular maturational process known as maturation (Eddy, 2006; Gervasi and Visconti, 2017 and references therein). The epididymis is an organ formed by a convoluted tubule that extends from the testis to the *vas deferens*. The mouse epididymis is anatomically organized into four regions known as initial segment, caput, corpus and cauda (Breton et al., 2016; Domeniconi et al., 2016). During epididymal maturation sperm migrate from the initial segment toward the cauda region, where they are stored until ejaculation occurs. It is known that immature caput sperm are immotile and unable to fertilize (Gervasi and Visconti, 2017).

Conversely, mature cauda sperm are progressively motile and acquire their fertilization competence after a process of capacitation in the female reproductive tract (Gervasi and Visconti, 2016). The acquisition of progressive motility during epididymal maturation has been linked to inactivation of both, the glycogen synthase kinase 3 (GSK3) and the serine/threonine protein phosphatase 1 gamma (PP1γ) (Vijayaraghavan et al., 1996; Somanath et al., 2004; Bhattacharjee et al., 2018). The Wnt signaling pathway has been recently proposed as a new molecular player involved in sperm maturation (Koch et al., 2015). In addition, the regulation of lipid mediators from the endocannabinoid system has been related to sperm acquisition of motility during maturation. A gradient of the endocannabinoid 2-arachidonoylglycerol (2-AG) in the epididymis prevents activation of motility in caput sperm through activation of cannabinoid receptor 1 (CB1) (Ricci et al., 2007; Cobellis et al., 2010). In human sperm, 2-AG inhibits the sperm calcium channel (CatSper), and its degradation allows calcium influx and activation of sperm motility (Miller et al., 2016). Despite the advances in this field of study, the mechanisms by which immature sperm acquire progressive motility and the ability to capacitate when incubated in proper conditions remain largely unknown.

The unique nature of sperm cells being transcriptionally and translationally silent (Diez-Sanchez et al., 2003) has led researchers to investigate post-translational modifications as key regulators of sperm function. Phosphorylation events, either in serine/threonine or in tyrosine residues, have been related to both, sperm maturation and capacitation (Buffone et al., 2014; Dacheux and Dacheux, 2014). Incubation of cauda sperm in a capacitation media supplemented with ${\text{HCO}}_{3}^{-}$ and BSA induces a rapid activation of protein kinase A (PKA) which phosphorylates several substrates in serine/threonine residues (Visconti et al., 1997; Wertheimer et al., 2013). This massive phosphorylation event is followed by an increase of protein phosphorylation in tyrosine residues that leads to acquisition of fertilization competence (Visconti et al., 1995a,b). Interestingly, tyrosine phosphorylation is not attainable in immature caput sperm regardless of the supporting media (Visconti et al., 1995a) even after the addition of permeable cAMP agonists.

In recent years, the addition of O-linked β-N-Acetylglucosamine (O-GlcNAc) to proteins in serine or threonine residues has been described as a new post-translational modification in various cellular types (Yang and Qian, 2017). Contrary to phosphorylation, mediated by several families of kinases and phosphatases, the turnover of O-GlcNAc is tightly regulated by only two well-conserved enzymes: uridine diphospho-N-acetylglucosamine:polypeptide β-N-acetylglucosaminyl transferase (O-GlcNAc transferase, OGT) and β-D-N-acetylglucosaminidase (O-GlcNAcase, OGA) (Hu et al., 2010). OGT is the enzyme that transfers O-GlcNAc from the donor substrate UDP-glucosamine to serine/threonine residues of proteins, and OGA is the enzyme that hydrolyzes this modification (Hart et al., 2007). It has been shown by generation of a knock-out mouse line that OGT is required for mouse embryonic development (Shafi et al., 2000), and by conditional mutagenesis, that OGT is essential for somatic cell function (O'Donnell et al., 2004). Generation of an OGA conditional knock-out model indicated that this enzyme is critical to maintain metabolic homeostasis and, animals lacking OGA died shortly after birth (Keembiyehetty et al., 2015). The interplay between O-GlcNAcylation and protein phosphorylation has been proposed as a mechanism that regulates cellular homeostasis with several levels of complexity (Hart et al., 2007; Mishra et al., 2011; Yang and Qian, 2017). In addition, it has been shown that OGT forms functional complexes with PP1γ in the brain (Wells et al., 2004). Considering that PP1γ activity is regulated during epididymal maturation (Vijayaraghavan et al., 1996), the interplay between O-GlcNAcylation and phosphorylation could be part of the mechanism by which caput sperm acquire progressive motility during their transit through the epididymis. There is still no evidence of the presence of this post-translational modification in sperm. Therefore, the aim of this work was first to characterize the O-GlcNAc system in male reproductive tissues; and second, to investigate the extent by which this post-translational modification is regulated in sperm during epididymal maturation. We report here that both, the transferase and the post-translational modification, are present in testis, epididymis, and sperm cells. The levels of O-GlcNAc were found to decrease in sperm during epididymal maturation. In addition, OGT and O-GlcNAcylated proteins also showed differences in their localization when sperm recovered from the caput and the cauda regions were compared. Altogether, these results indicate that sperm O-GlcNAcylation is regulated during mouse sperm maturation and suggest a functional role for this post-translational modification.

## Results

### Characterization of O-GlcNAcylation in mouse testis

**Figure 1:**
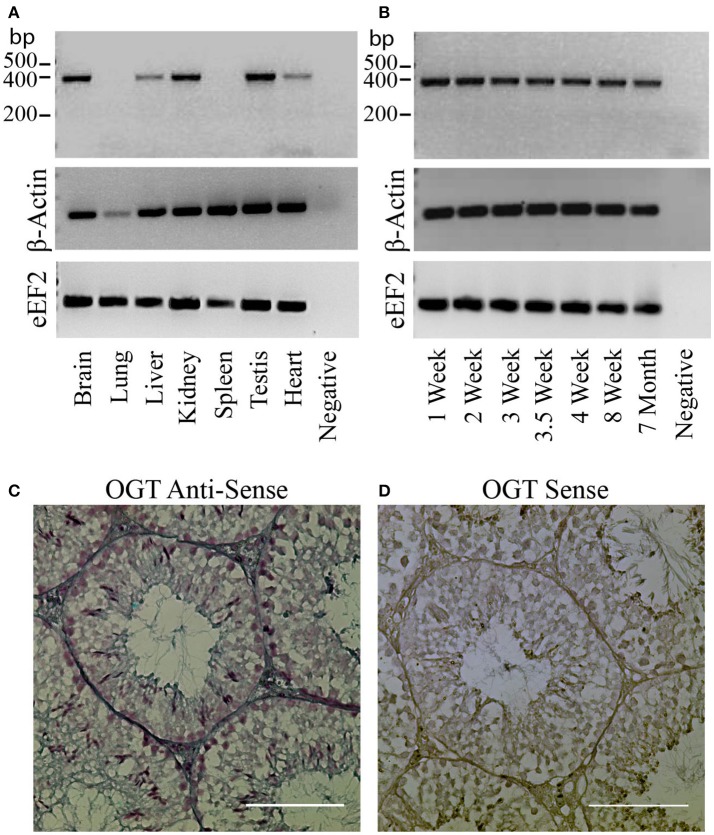
OGT expression in mouse testis. **(A)** Analysis of OGT mRNA expression by RT-PCR in various adult mouse tissues: brain, lung, liver, kidney, spleen, testis, and heart. β-actin and eEF2 were used as loading control. *N* = 3 **(B)** Analysis of OGT mRNA expression by RT-PCR during post-natal testis development: 1 week-, 2 week-, 3 week-, 3.5 week-, 4 week-, 8 week-, and 7 month-old mice. β-actin and eEF2 were used as loading control. *N* = 3. **(C)** *In-situ* hybridization of OGT (anti-sense probe) in adult mouse testis. **(D)** Sense OGT probe used as negative control. Images are representative of three independent experiments. Scale bar = 100 μm.

The protein OGT (Uniprot ref # Q8CGY8, IUPAC EC 2.4.1.255) is encoded by the gene Ogt (ID: 108155). We used RT-PCR to compare the mRNA levels of the transferase OGT present in testis with the ones expressed in other tissues. OGT mRNA was found to be expressed in adult mouse brain, liver, kidney, heart, and testis (Figure 1A). The RT-PCR analyses at various postnatal ages indicated that OGT expression levels did not variate between testes that present cells at diverse gametogenic stages (Figure 1B). Because other testicular cell types could be expressing OGT, *In-situ* hybridization experiments were performed to complement the RT-PCR results. *In-situ* hybridization results indicated that OGT transcripts are present in all germ cell types in the adult male testis (Figure 1C). As a negative control, mouse testes were incubated with a sense probe and no staining was observed (Figure 1D).

**Figure 2:**
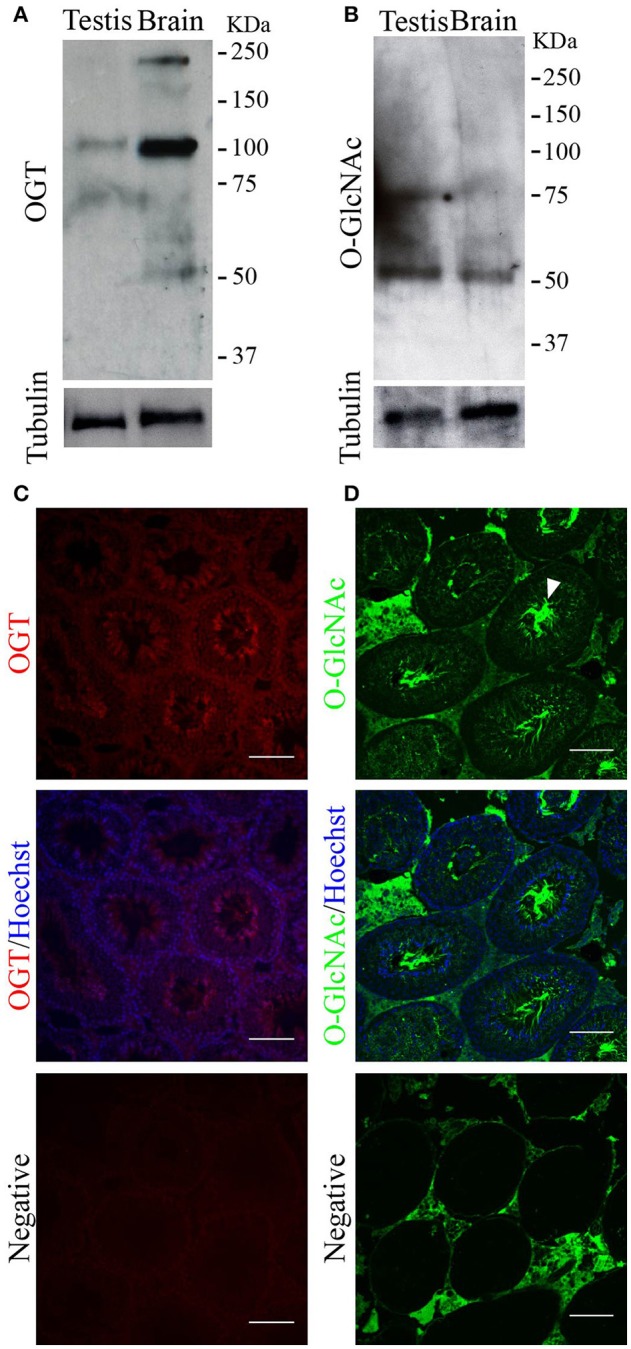
OGT and O-GlcNAcylated proteins in mouse testis. **(A)** Western blotting analysis of OGT in adult testis. Rat brain was used as positive control. Membrane stripping and re-probing with anti-tubulin antibody was used as loading control. *N* = 4 **(B)** Western blotting analysis of O-GlcNAcylated proteins (anti-O-GlcNAc antibody, clone CTD110.6) in adult testis. Rat brain was used as positive control. Membrane stripping and re-probing with anti-tubulin antibody was used as loading control. *N* = 4 **(C)** Immunofluorescence analysis indicating OGT localization in adult testis (upper panel). OGT signal merged with Hoechst nuclear staining (medium panel). Negative control was performed by incubation with secondary antibody alone (lower panel). *N* = 3 **(D)** Immunofluorescence analysis indicating O-GlcNAc (anti-O-GlcNAc antibody, clone CTD110.6) localization in adult testis. Arrowhead indicates the sperm flagella. O-GlcNAc staining merged with Hoechst nuclear staining (medium panel). Negative control was performed by incubation with secondary antibody alone (lower panel). Images are representative of three independent experiments. Scale bar = 100 μm.

The next step was to determine the presence of the protein OGT and of the post-translational modification O-GlcNAcylation in testis by Western blotting and immunofluorescence. Our results showed that both, a ~110 KDa band corresponding to OGT (Figure 2A), and a band corresponding to an O-GlcNAcylated protein (Figure 2B) were present in mouse testis extracts. OGT was found in all germ cell types during spermatogenesis (Figure 2C). In addition, the localization of O-GlcNAcylated proteins was also observed in all germ cell types within the testis with high intensity in the sperm flagella (Figure 2D). These results indicate that OGT is present in mouse testis, and that O-GlcNAcylation of testicular proteins occurs during all the phases of spermatogenesis. Negative controls showed some levels of unspecific fluorescence in the connective tissue with undetectable unspecific signal within the seminiferous tubules (Figures 2C,D lower panel).

### O-GlcNAcylation in mouse epididymal sperm

**Figure 3:**
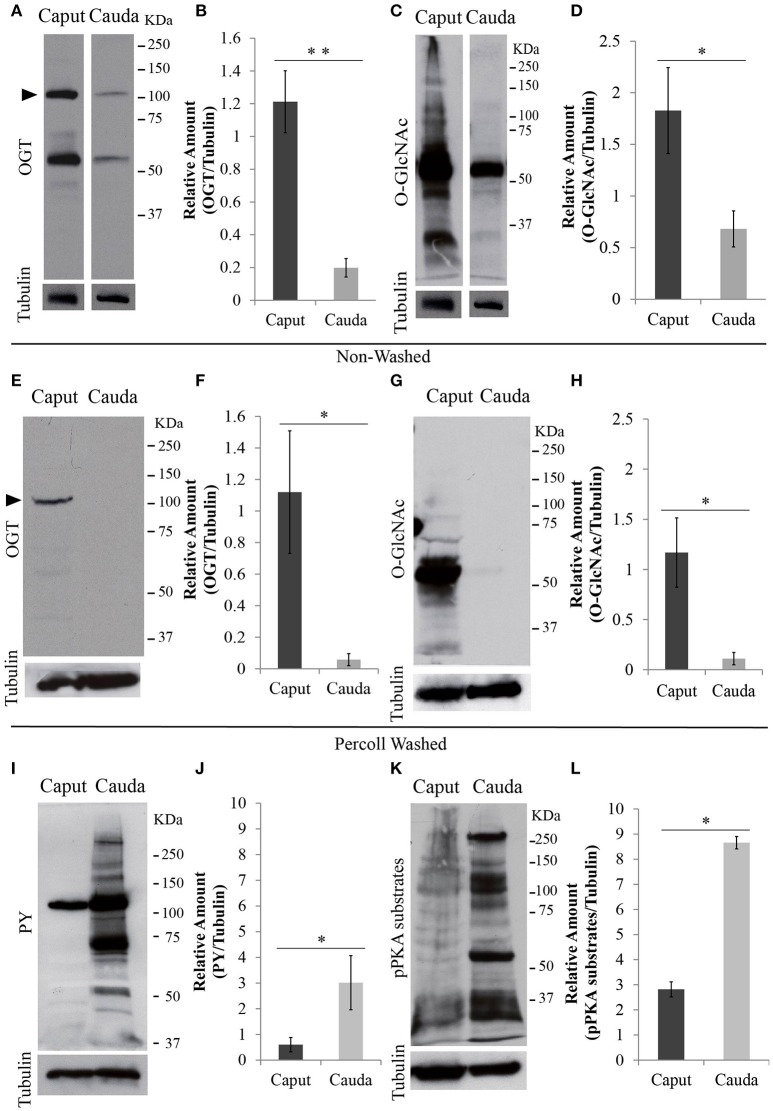
Protein analyses of OGT and post-translational modifications in epididymal sperm. Sperm were collected from caput or cauda epididymis and proteins were extracted and separated by SDS-PAGE. **(A)** Western blotting of OGT from unwashed collection of caput and cauda sperm. Membranes were stripped and re-probed with anti-tubulin antibody to evaluate equal loading. *N* = 4 **(B)** Graph indicating the optical densitometry ratio between OGT and tubulin. Statistical significance between groups is indicated, ^**^p < 0.01. **(C)** Western blotting of O-GlcNAcylated proteins (anti-O-GlcNAc antibody, clone CTD110.6) from unwashed collection of caput and cauda sperm. Membranes were stripped and re-probed with anti-tubulin antibody to evaluate equal loading. *N* = 4 **(D)** Graph indicating the optical densitometry ratio between O-GlcNAcylated proteins and tubulin. Statistical significance between groups is indicated, ^*^p < 0.05. **(E)** Western blotting of OGT from Percoll-washed caput and cauda sperm. Membrane stripping and re-probing with tubulin antibody was used as loading control. *N* = 3. **(F)** Graph indicating the optical densitometry ratio between OGT and tubulin. Statistical significance between groups is indicated, ^*^p < 0.05. **(G)** Western blotting of O-GlcNAcylated proteins (anti-O-GlcNAc antibody, clone CTD110.6) in Percoll-washed caput and cauda sperm. Membranes were stripped and re-probed with anti-tubulin antibody to evaluate equal loading. *N* = 3. **(H)** Graph indicating the optical densitometry ratio between O-GlcNAcylated proteins and tubulin. Statistical significance between groups is indicated, ^*^p < 0.05. **(I)** Western blotting of tyrosine phosphorylation (PY) after 60-min-capacitation of unwashed caput and cauda sperm. Membranes were stripped and re-probed with anti-tubulin antibody to evaluate equal loading. *N* = 4 **(J)** Graph indicating the optical densitometry ratio between PY and tubulin. Statistical significance between groups is indicated, ^*^p < 0.05. **(K)** Western blotting of phosphorylated protein kinase A substrates (pPKA substrates) after 60-min-capacitation of unwashed caput and cauda sperm. Membranes were stripped and re-probed with anti-tubulin antibody to evaluate equal loading. *N* = 4 **(L)** Graph indicating the optical densitometry ratio between p-PKA and tubulin. Statistical significance between groups is indicated, ^*^p < 0.05.

After leaving the testis, sperm undergo maturation as part of their transit from the caput to the cauda epididymis. Similar to testicular sperm, OGT is present in caput sperm. However, the amount of OGT was significantly decreased in cauda sperm (Figures 3A,B). Besides the predicted ~110 KDa protein detected by the anti-OGT antibody, a second band at ~60 KDa was recognized. This could be a product of OGT degradation. In addition, caput sperm contained high levels of O-GlcNAcylated proteins and this post-translational modification was significantly reduced in cauda spermatozoa (Figures 3C,D). Similar differences in protein O-GlcNAcylation between caput and cauda sperm were found when an alternative specific O-GlcNAc antibody (clone RL2) was used for immunodetection (Supplementary Figure 1A).

Contrary to cauda sperm collection resulting in almost pure sperm populations, caput sperm suspensions typically contain other cell types and debris. Therefore, to assure that the OGT and O-GlcNAc signals were of sperm origin, each of the sperm samples obtained from caput and cauda were purified using a Percoll wash as explained in the methods section. Western blotting of Percoll-washed sperm also indicated higher levels of OGT (Figures 3E,F) and O-GlcNAc modification (Figures 3G,H) in sperm from caput epididymis when compared to those from cauda.

O-GlcNAc modifications occur in serine/threonine amino acids and have been postulated to block phosphorylation of these residues and consequently counteract phosphorylation pathways (Hu et al., 2010). Contrary to cauda sperm, caput sperm are not capable to undergo capacitation-induced increase in tyrosine phosphorylation (Visconti et al., 1995a). Our current observations confirmed those findings (Figures 3I,J). Although O-GlcNAc does not occur on tyrosine residues, tyrosine phosphorylation during cauda sperm capacitation is downstream of phosphorylation cascades involving PKA, a serine/threonine kinase. Interestingly, PKA-dependent phosphorylation was also blocked in caput epididymal sperm (Figures 3K,L). Currently, because of the complex protein pattern of both PKA-dependent phosphorylation and O-GlcNAcylation, it is not possible to know the extent by which these post-translational modifications occur in the same proteins and residues.

**Figure 4:**
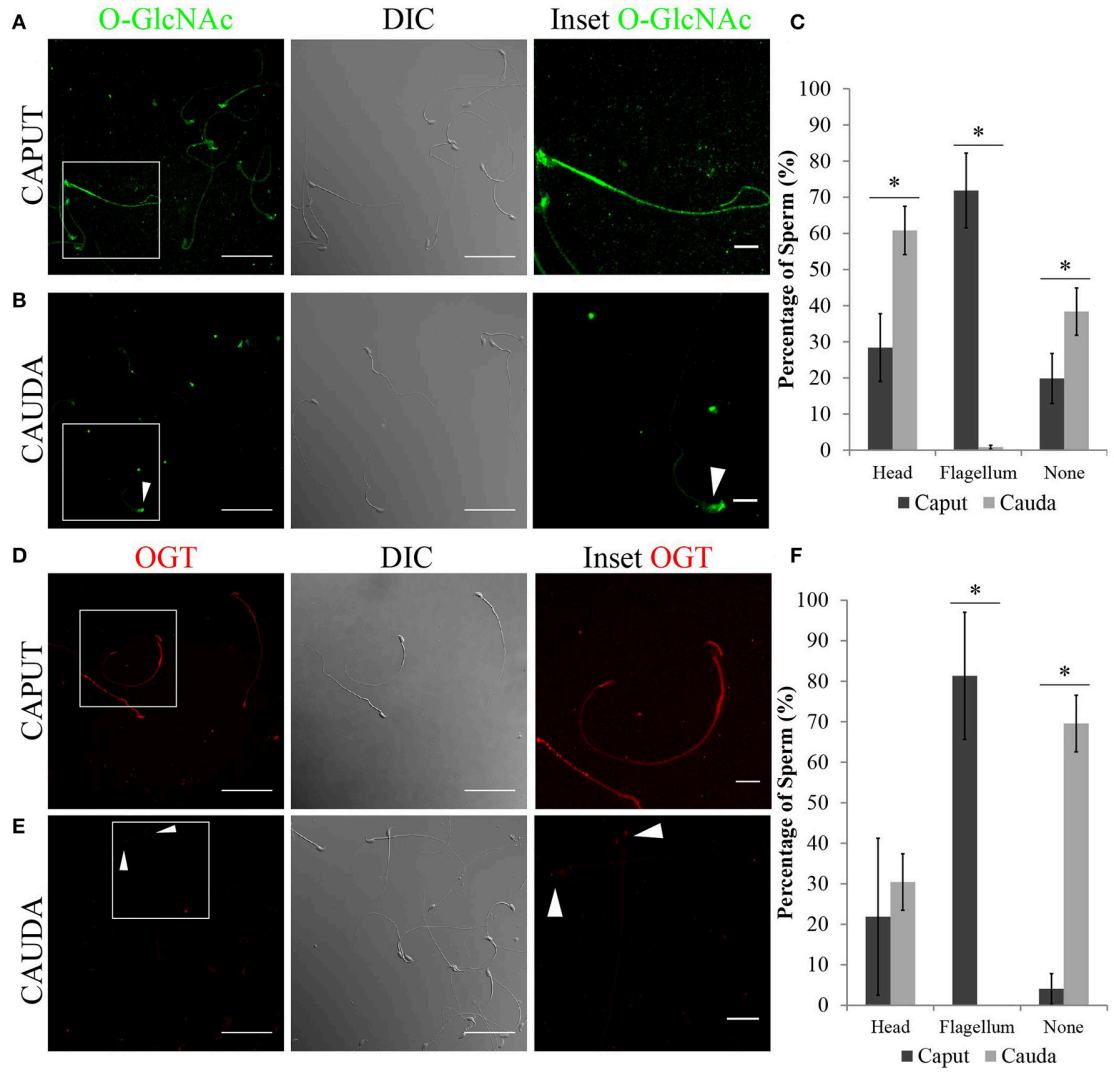
Localization of O-GlcNAcylated proteins and OGT in epididymal sperm by immunofluorescence. **(A)** Localization of O-GlcNAcylated proteins (O-GlcNAc, green) in sperm recovered from the caput region (left panel), scale bar 50 μm. DIC image of the same field of view (middle panel). O-GlcNAc inset (right panel), scale bar = 10 μm. **(B)** Localization of O-GlcNAcylated proteins (O-GlcNAc, green) in sperm recovered from the cauda region (left panel), scale bar = 50 μm. DIC image of the same field of view (middle panel). O-GlcNAc inset (right panel), scale bar = 10 μm. Arrowhead indicates the sperm head. **(C)** Quantification of O-GlcNAc immunofluorescences shown in **(A,B)**. A total of 425 caput and 671 cauda sperm from six independent experiments were counted. Statistical significance between groups is indicated, ^*^p < 0.05. **(D)** Localization of OGT (red) in sperm recovered from the caput region (right panel), scale bar = 50 μm. DIC image of the same field of view (middle panel). OGT inset (right panel), scale bar = 10 μm. **(E)** Localization of OGT (red) in sperm recovered from the cauda region (left panel), scale bar = 50 μm. DIC image of the same field of view (middle panel). OGT inset (right panel), scale bar = 10 μm. Arrowheads indicate the sperm head. **(F)** Quantification of OGT immunofluorescences shown in **(D**,**E)**. A total of 350 caput and 915 cauda sperm from four independent experiments were counted. Statistical significance between groups is indicated, ^*^p < 0.05.

Next, the localization of O-GlcNAcylated proteins in sperm recovered from caput and cauda epididymis was evaluated by immunofluorescence. Caput sperm presented O-GlcNAcylated proteins in the head and throughout the flagellum (Figure 4A), while in cauda sperm the signal was absent or restricted to the head region (Figure 4B). The different patterns found for O-GlcNAcylated proteins (head, flagellum or absent) were quantified, and the results indicated a significant difference between caput and cauda sperm (Figure 4C). The differential localization of O-GlcNAcylated proteins in caput and cauda sperm was also detected by immunofluorescence using the alternative O-GlcNAc specific antibody clone RL2 (Supplementary Figures 1B,C). These results are in agreement with the Western analyses, shown in Figure 3, and indicate that the levels of O-GlcNAcylation in sperm decrease during epididymal maturation.

As OGT is the only known enzyme that catalyzes the addition of O-GlcNAc to proteins, the localization of OGT was also evaluated. Coincidently with O-GlcNAc localization, OGT was found mainly localized to the flagellum of caput sperm (Figure 4D), while it was absent or restricted to the head of cauda sperm (Figure 4E). The differences in localization of OGT between caput and cauda sperm were quantified and the results show that a high percentage of cauda sperm have undetectable levels of OGT (Figure 4F). Overall, our results point to a decrease in the levels of protein O-GlcNAcylation during sperm maturation which is consistent with the loss of OGT during this process.

### OGT and O-GlcNAcylated proteins in mouse epididymis

**Figure 5:**
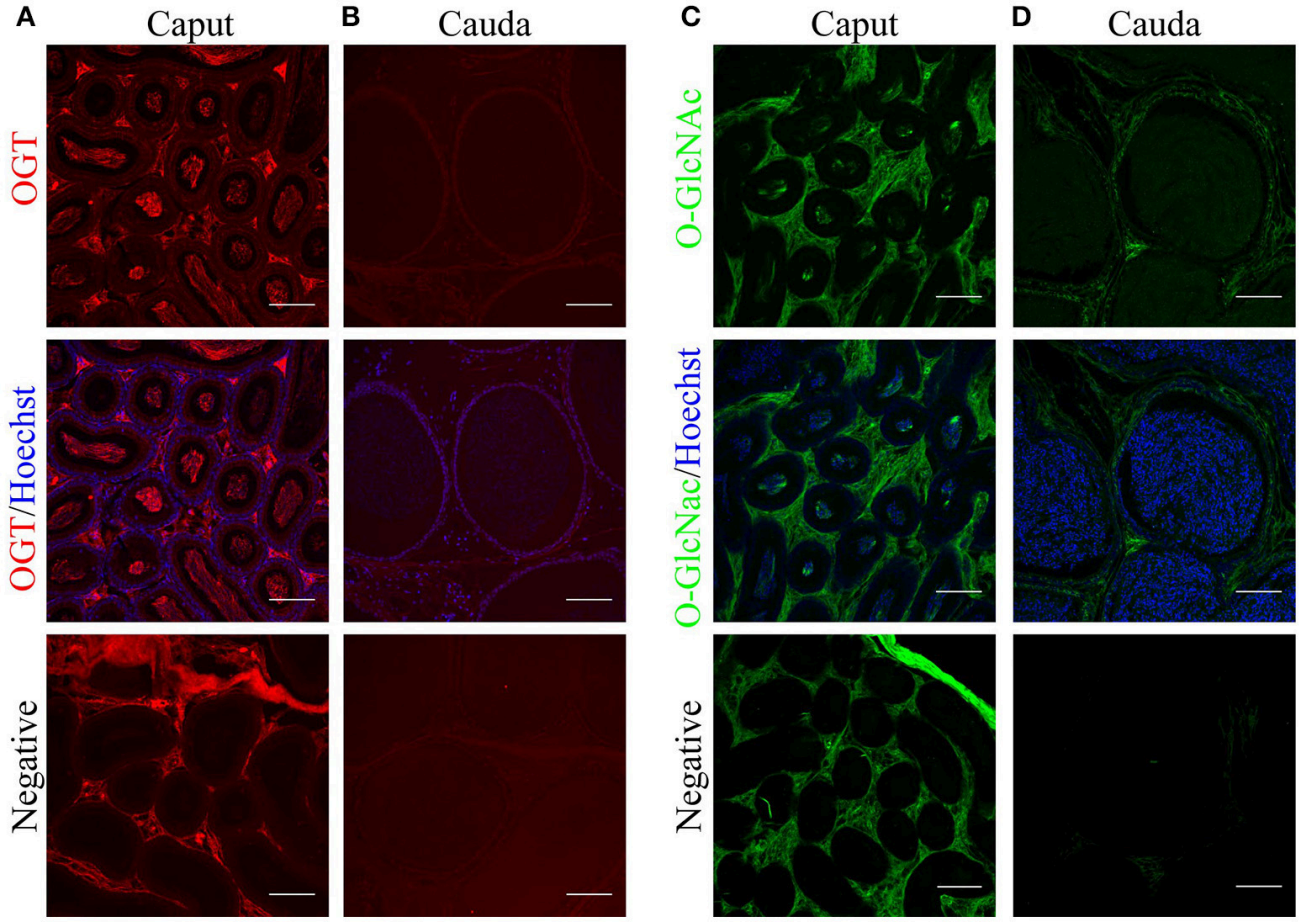
Localization of O-GlcNAcylated proteins and OGT in mouse epididymal tissue by immunofluorescence. **(A)** Localization of OGT (red) in caput epididymal sections (upper panel). OGT signal was merged with Hoechst nuclear staining (medium panel). Negative control was performed by incubation with secondary antibody alone (lower panel). **(B)** Localization of OGT (red) in cauda epididymal sections (upper panel). OGT signal merged with Hoechst nuclear staining (medium panel). Negative control was performed by incubation with secondary antibody alone (lower panel). **(C)** Localization of O-GlcNAcylated proteins (anti-O-GlcNAc antibody clone CTD110.6, green) in caput epididymal sections (upper panel). O-GlcNAc signal merged with Hoechst nuclear staining (medium panel). Negative control was performed by incubation with secondary antibody alone (lower panel). **(D)** Localization of O-GlcNAcylated proteins (anti-O-GlcNAc antibody clone CTD110.6, green) in cauda epididymal sections (upper panel). O-GlcNAc signal merged with Hoechst nuclear staining (medium panel). Negative control was performed by incubation with secondary antibody alone (lower panel). Images are representative of three independent experiments. Scale bars = 100 μm.

Sperm maturation occurs in the male epididymis. Therefore, we investigated OGT and O-GlcNAc localization in the mouse epididymis by immunofluorescence. Caput epididymis presented high levels of OGT in the luminal epithelium and in the center of the tubules where the sperm cells are located (Figure 5A). In the cauda epididymal epithelium, low levels of OGT staining were found. Similar to the results shown above, OGT was lost in cauda spermatozoa (Figure 5B). When O-GlcNAc was evaluated in caput epididymis, this post-translational modification was absent from the epithelium and only localized to the center of the tubules where the sperm are located (Figure 5C). In contrast, the cauda epididymis showed O-GlcNAcylated proteins in the epithelium, suggesting that this pathway remains partially active in these cells. No O-GlcNAcylation signal was detected in cauda sperm (Figure 5D). Negative controls displayed some unspecific background in the connective tissue but neither in sperm nor in the epididymal epithelium. Altogether, these data indicate that there is a differential localization of OGT and the occurrence of O-GlcNAcylation between caput and cauda epididymis. In addition, these results support our findings indicating that O-GlcNAc is regulated in sperm during epididymal maturation.

## Discussion

Several post-translational modifications have been shown to change in sperm during epididymal transit (Gervasi and Visconti, 2017). Being transcriptionally and translationally inactive, these changes are particularly relevant in sperm. The proteome is limited by a fixed number of genes, but protein functionality can be amplified by post-translational modifications that regulate cellular processes (Walsh et al., 2005). Phosphorylation, acetylation, methylation, and ubiquitylation can be found amongst the most widely studied protein modifications. In the last decades, O-GlcNAcylation of proteins in serine or threonine residues has emerged as a novel mechanism for protein regulation (Torres and Hart, 1984; Holt and Hart, 1986). This post-translational modification involves a unique glycosylation event in which a single O-linked β-N-acetylglucosamine (O-GlcNAc) moiety is actively transferred from the donor UDP-GlcNAc to a hydroxyl group of the recipient amino acid (Hart et al., 2007; Yang and Qian, 2017). OGT is the enzyme responsible for the transfer of O-GlcNAc to proteins (Haltiwanger et al., 1992), and OGA is the enzyme responsible for O-GlcNAc hydrolisys (Gao et al., 2001). The occurrence of O-GlcNAcylation in sperm and the male reproductive tract has not been previously reported. In the present work, we studied this novel system and evaluated its association to sperm maturation.

RT-PCR expression analyses indicated that OGT transcripts are found in the testis as well as in other mouse tissues. These results are in agreement with the OGT expression analysis conducted by Shafi and coworkers (Shafi et al., 2000). In addition, we found that the OGT mRNA and protein are present in all phases of spermatogenesis, and that O-GlcNAcylated proteins in the mouse testis are found in all germ cell types from spermatogonia to testicular sperm. These levels of O-GlcNAcylation suggest that this post-translational modification is functional during spermatogenesis.

During epididymal maturation, sperm undergo a series of changes at the molecular level that induce their ability to move progressively and the potential to become fertilization-competent after capacitation (Gervasi and Visconti, 2017). The molecular basis of sperm maturation is largely unknown. However, the inactivation of PP1γ is required for the acquisition of progressive motility in sperm (Vijayaraghavan et al., 1996, 2007). Interestingly, in the brain, the catalytic subunit of PP1γ forms functional complexes with OGT (Wells et al., 2004). Considering that PP1γ and OGT target serine/threonine sites of proteins, the reported elevated activity of PP1γ in caput sperm is consistent with the increased O-GlcNAcylation of proteins found.

Due to the impossibility of reproducing sperm maturation in *in vitro* models, the study of this process relies on the comparison between sperm recovered from caput and cauda regions. It has been proposed that in caput sperm PP1γ remains active by indirect action of GSK3 (Vijayaraghavan et al., 1996) and, that during epididymal maturation, GSK3 is inactivated by phosphorylation in serine residues. The latter is indicated by the increase in phosphorylated GSK3 in cauda sperm when compared with caput (Somanath et al., 2004). Several protein kinases have been proposed to phosphorylate and inactivate GSK3, Akt being one of them (Vadnais et al., 2013). In addition, it has been shown that Akt undergoes O-GlcNAcylation in other cell types (Vosseller et al., 2002), and that this post-translational modification decreases Akt activity (Wang et al., 2012). Interestingly, we found decreased levels of O-GlcNAcylated proteins in mature cauda sperm. One possible mechanism for the acquisition of sperm motility in cauda sperm is an increase of Akt activity due to decreased Akt O-GlcNAcylation. Further experiments are needed to test this hypothesis.

After gaining basal motility, when mature cauda sperm are exposed to capacitation conditions, a cascade of signaling events that leads to acquisition of sperm fertilization ability is activated (Gervasi and Visconti, 2016). Fast activation of PKA and phosphorylation of PKA substrates is followed by a later increase in tyrosine phosphorylation (Visconti, 2009). Contrary to mature cauda sperm, immature caput sperm are unable to undergo phosphorylation on tyrosine residues when incubated in conditions that support capacitation (Visconti et al., 1995a). In the present work, we confirmed that immature caput sperm are not able to display an increase in tyrosine phosphorylation. In addition, we found that PKA-dependent phosphorylation is also decreased in caput sperm when compared to mature sperm. PKA phosphorylates its substrates in serine/threonine residues; therefore, there is a possible competition for these sites between PKA and OGT. Localization of the catalytic subunit of PKA to the sperm flagellum in mature sperm (Wertheimer et al., 2013) coincides with the localization of OGT found in immature caput sperm. Then, OGT could be targeting PKA substrates and preventing their phosphorylation by catalyzing O-GlcNAcylation of the available amino acid residues. In this sense, caput OGT induces O-GlcNAcylation of proteins in caput sperm, and therefore might be preventing phosphorylation of PKA substrates and the activation of the sperm capacitation pathway that triggers the acquisition of fertilization competence. Consistent with this hypothesis, we found the OGT enzyme is mostly absent from cauda sperm, and the levels of O-GlcNAcylated proteins were greatly reduced. This would increase the availability of sites for PKA phosphorylation and activation of the capacitation pathway when sperm are exposed to the proper stimuli.

Differential gene expression and luminal concentrations of ions and endocannabinoids in the different regions of the segmented epididymis are essential to coordinate sperm maturation (Cobellis et al., 2010; Belleannée et al., 2012a,b; Björkgren et al., 2015). Therefore, the differential expression of the O-GlcNAc transferase OGT found between caput and cauda luminal epithelium is consistent with a functional difference of these epididymal regions. The mechanisms by which OGT is lost from sperm during epididymal transit is still unknown. Most reports indicate that proteins are incorporated to sperm during maturation (reviewed in Gervasi and Visconti, 2017), however the protein dicarbonyl L-xylulose reductase (DCXR) has been reported to be selectively removed from bovine sperm during maturation (Akintayo et al., 2015). Our data suggest that OGT is lost from the sperm flagellum during transit through the epididymis, and further investigations are necessary to determine whether it is due to the specific degradation of this transferase.

Protein O-GlcNAcylation is regulated by the fine-tuned activity of the enzymes OGT and OGA (Hart et al., 2007). The lack of either of these enzymes disrupts cell homeostasis and function (Shafi et al., 2000; O'Donnell et al., 2004; Keembiyehetty et al., 2015). Therefore, in addition to OGT, it is important to take into consideration the possible modulation of OGA during sperm maturation. Future investigations regarding OGA are required to fulfill this need.

**Figure 6:**
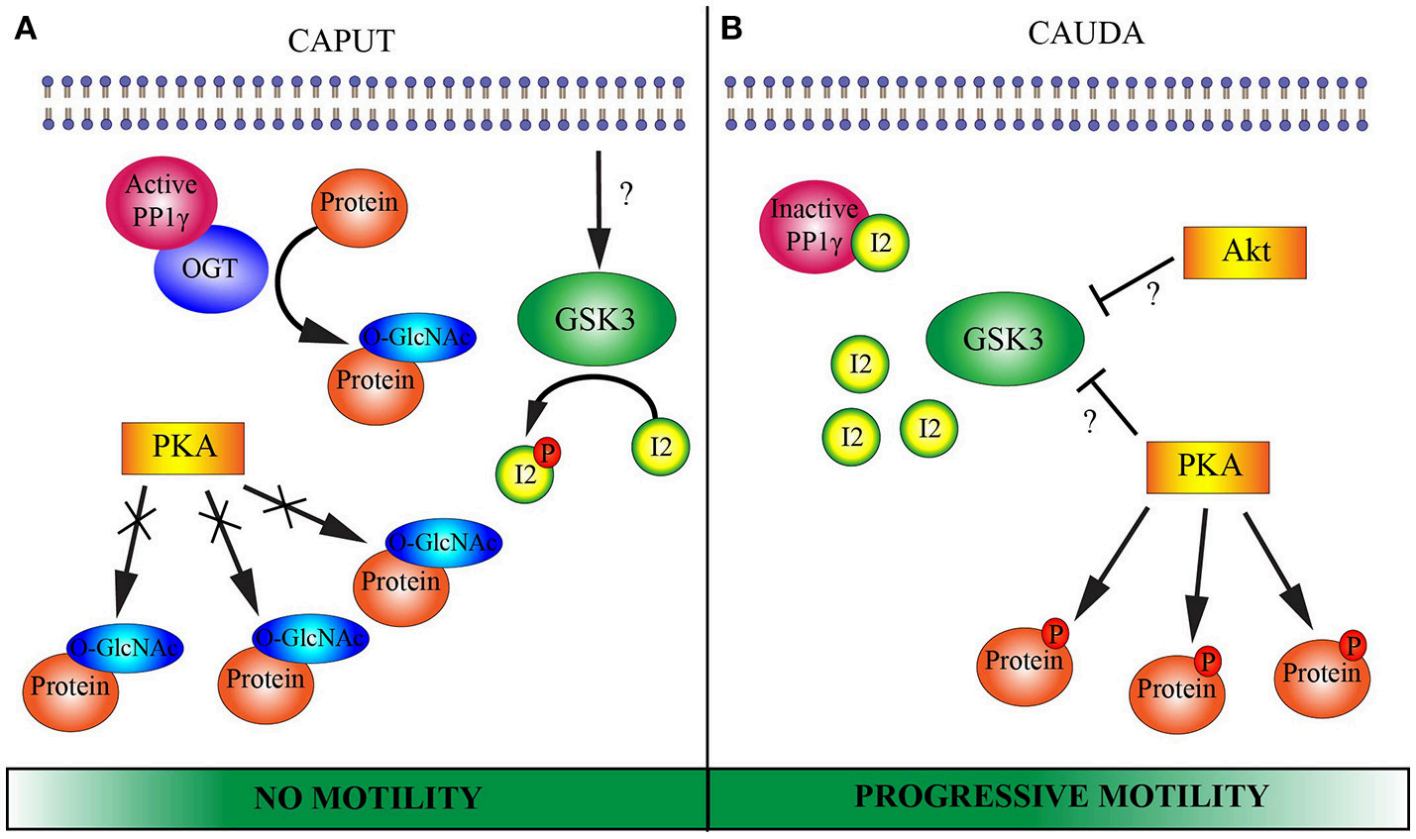
Proposed model of putative molecular pathways involved in epididymal sperm maturation**. (A)** CAPUT: Glycogen synthase kinase 3 (GSK3) is active and phosphorylates protein inhibitor 2 (I2). Once phosphorylated, I2 is not able to bind to and inactivate the serine/threonine phosphatase PP1γ. OGT potentially interacts with PP1γ which would remove phosphates from serine/threonine residues and render them available for OGT. OGT catalyzes O-GlcNAcylation in serine/threonine residues of several proteins. This O-GlcNAcylation potentially blocks phosphorylation sites for protein kinase A (PKA). Caput sperm lack motility. **(B)** CAUDA: GSK3 is phosphorylated on serine residues and rendered inactive by an unidentified serine/threonine kinase. Among the proposed kinases involved in GSK3 phosphorylation and inactivation are PKA and RAC-alpha protein kinase (Akt). Due to GSK3 inactivation, protein I2 can no longer undergo phosphorylation and consequently binds to PP1γ, inactivating its catalytic activity. OGT is absent from cauda sperm and O-GlcNAcylation of proteins is lost. Then, serine/threonine sites are available for phosphorylation by PKA. Together, inactivation of PP1γ leads to the ability of the sperm cell to move progressively and PKA can phosphorylate its substrates when exposed to an appropriate medium. Note that the Wnt and 2-AG pathways mentioned in the introduction have been excluded from the schematic model with the goal of simplifying the understanding of the molecular pathways.

The present study introduces O-GlcNAcylation as a novel molecular player that participates in epididymal sperm maturation. The drastic changes in O-GlcNAcylation of spermatic proteins during maturation could be part of the still unknown molecular mechanisms that regulate the acquisition of fertilization competence. A model illustrating the incorporation of OGT and O-GlcNAc to the established pathways is presented in Figure 6. Our work raises further questions related to sperm maturation. More work is needed to investigate the possible interaction between OGT and PP1γ in immature sperm, and its connection to the acquisition of sperm motility.

## Methods

### Animals

Mouse sperm samples were collected from male CD1 retired breeders (Charles River Laboratories, Wilmington, MA, USA). Animal care and use of experimental animals was conducted in accordance with specific guidelines and standards dictated by the Office of Laboratory Animal Welfare (OLAW) and approved by the Institutional Animal Use and Care Committee (IACUC), University of Massachusetts-Amherst (Protocol #2016-0026).

### Media

The non-capacitation medium used for sperm was Hepes-based, modified Toyoda-Yokoyama-Hosi (m-TYH) medium, consisting of (in millimolar): 119.37 NaCl, 4.78 KCl, 1.19 KH_2_PO_4_, 1.19 MgSO_4_, 5.56 glucose, 1.71 CaCl_2_, 20 HEPES, 0.51 Na-pyruvate, 10 μg/mL gentamicin, 0.0006 % phenol red at pH 7.2 to pH 7.4. Capacitating m-TYH was prepared by supplementing non-capacitation media with 25 mM NaHCO_3_ and 5 mg/mL of bovine serum albumin (BSA, Sigma cat # A0281, St. Louis, MO) to the m-TYH at pH 7.2 to 7.4.

### Sperm sample collection

Caput epididymis sperm were obtained by squeezing the tissue in non-capacitating m-TYH media approximately 10–15 times. Cauda epididymis sperm were obtained by the “swim-out” method using the same non-capacitating m-TYH media. Briefly, cauda epididymides were dissected, and three incisions were made with fine scissors prior placing the tissue into 1 ml of m-TYH media. Sperm were allowed to swim-out for 10 min at 37°C, and then the epididymides were removed from the sperm suspension. In experiments in which phosphorylation by PKA and tyrosine phosphorylation was investigated, sperm were incubated for 60 min in capacitation media. Otherwise, sperm were used directly after recovery from tissue for further analysis.

### Sperm purification

Caput and cauda epididymal sperm were purified by modification of a method previously described (Vadnais et al., 2013). Briefly, samples were centrifuged at 600 × g for 20 min in a 35% Percoll (Sigma, cat # P1644, St. Louis, MO) in PBS column. After centrifugation, purified sperm found in the pellet were resuspended in PBS and centrifuged at 800 × g for 10 min at room temperature. The sperm pellets were resuspended in m-TYH and processed for Western blotting.

### Reverse-transcriptase-PCR (RT-PCR)

Tissues from adult male mice were collected: brain, lung, liver, spleen, kidney, heart, and testis (1-week to 8-month old); then homogenized and total RNA was extracted using High Pure RNA Isolation Kit (Roche, Indianapolis, IN). Total RNA concentration was validated and measured using a Nanodrop Spectrophotometer (BioDrop, Cambridge, UK). cDNA was synthesized from RNA samples using iScript^TM^ cDNA Synthesis Kit (Bio-Rad, Hercules, CA) in 20 μL reaction volume. Starting amount of total RNA used was 500 ng. Reverse transcriptase PCR was performed with the following oligonucleotide primers: *OGT* (anti-sense: 5′-TAATACGACTCACTATAGGGTGTCACCTGCTGCTACCAAG- 3′); *OGT* (sense: 5′-TAATACGACTCACTATAGGGTTAGCTGAGTTGGCACATCG-3′); β-Actin (anti-sense: 5′-GACGATGCTCCCCGGGCTGTATTC-3′); β-Actin (sense: 5′- TCTCTTGCTCTGGGCCTCGTCACC-3′); eEF2 (sense: 5′-GCGTGCCAAGAAAGTAGAGG-3′); and eEF2 (anti-sense: 5′-GGGATGGTAAGTGGATGGTG-3′) (Integrated DNA Technologies). Amplifications were performed using Taq DNA polymerase enzyme master mix (Affimetrix, Santa Clara, CA). For OGT and β-Actin PCR were performed as follows: 98°C for 1 min (initial denaturation) and 35 cycles at 95°C for 30 s, 56°C for 30 s, 72°C for 30 s and 72°C for 2 min. For eEF2 PCR was performed as follows: 95°C for 2 min (initial denaturation) and 35 cycles at 95°C for 30 s, 55.2°C for 30 s, 72°C for 30 s and 72°C for 2 min. PCR products were separated on a 2% (w/v) agarose gel, stained with ethidium bromide and detected under UV light.

### Tissue collection and preparation for immunofluorescence

Mouse testis and epididymal tissue were dissected and fixed in 4% paraformaldehyde (EMS, Hatfield, PA) in PBS overnight at 4°C. Then, progressive dehydration was performed by incubation in increasing concentrations of methanol (25, 50, 75, and 100%), and tissues were left overnight in 100% methanol at −20°C. The following day, the tissues were incubated in 100% xylene for 1 h at room temperature, proceeded by a second replicate incubation of xylene and then an overnight incubation in paraffin at 37°C. Blocks of paraffin-embedded tissue were then prepared and left to harden at room temperature before storage at 4°C. Tissues were sectioned at 7–9 μm-thick slices, lifted onto Superfrost Plus positively charged glass slides (Fisherbrand, Waltham, MA) and left to dry at 37°C overnight before being stored at −20°C. Rehydration was performed by first incubation with 100% xylene for 15 min at room temperature, followed by a second 100% xylene incubation, followed by incubation in decreasing ethanol concentrations in (100, 95, and 70%).

### *In-situ* hybridization

Mouse testis sections were prepared and rehydrated as described above. Sections were then permeabilized with Proteinase-K for 13 min. After slides were washed three times for 5 min with PBS-T (0.1% Tween-20 in PBS prepared with DEPC-Water), fixation with 4% paraformaldehyde and 0.2% glutaraldehyde for 20 min at room temperature was performed. Then, slides were washed for 5 min with PBS-T. Sections were then incubated with pre-heated hybridization buffer (50% Formamide, 1% SDS, 5X SSC (3M NaCl, 0.3M Na_2·_Citrate_2·_H_2_O), 5μg/mL Heparin, 50 μg/mL Yeast tRNA) and placed into an air-tight incubation chamber for 1 h at 65°C. Followed, by incubation with hybridization buffer and 600 ng/μL of OGT sense or anti-sense probe in an air-tight incubation chamber for 16 h at 65°C. Slides were then incubated twice with a di-formamide solution (2% 5X SSC, 0.1% Tween-20) for 30 min at 65°C. Sections were then washed three times for 5 min at room temperature with 5 mg/mL of Levamisole in PBS-T, proceeded another three times for 15 min at room temperature with the same media. Sections were then blocked with fetal goat serum in 10% BM blocking reagent for 30 min at room temperature, preceded with anti-digoxigenin block (1:500) for 1 h at room temperature. Another set of washes with 5 mg/mL of Levamisole in PBS-T were performed as described before washing with NTMT media (0.1% Tween-20, 5 M NaCl, 1 M MgCl, 1 M Tris at pH 9.5) for 5 min at room temperature. Slides were then stained with BM-purple-AP substrate (Roche, Indianapolis, IN) until desired staining was achieved. Once stained, slides were washed with 0.5 M EDTA three times for 5 min at room temperature. Then, fixation with 4% paraformaldehyde and 0.2% glutaraldehyde for 20 min at room temperature was performed, followed by washes with increasing ethanol concentrations (70, 90, and 100%) before a final xylene wash for 10 min at room temperature. Slides were then mounted with Cytoseal-60 (Richard-Allan Scientific, San Diego, CA). Brightfield images were taken using a 20X objective (Nikon, PlanApo, NA 0.75) in an inverted microscope (Nikon Eclipse T300) equipped with a RGB illumination system and an Andor Zyla camera.

### Whole testis protein extraction

Whole testis samples were collected and homogenized in ice-cold RIPA buffer (50 mM Tris-Cl pH 7.4, 150 mM NaCl, 10% glycerol, 0.1% SDS, 1% Triton X-100, 0.5% deoxycholate) supplemented with 1X protease cocktail inhibitors (Roche, Indianapolis, IN), 20 mM β-glycerophosphate, 10 mM Na_3_VO_4_. Samples were then placed on ice and vortexed every 5 min for 30 min, followed by centrifugation at 10,000 × g for 5 min at 4°C. After centrifugation, supernatant was transferred to fresh tubes and protein determination was done by the Bradford method (Bradford, 1976). 100 μg of protein were re-suspended in SDS-sample buffer (Laemmli, 1970), supplemented with 5% β-mercaptoethanol, boiled for 4 min and loaded in 8% SDS/PAGE. Analysis of proteins by Western blotting was performed as mentioned below.

### SDS/PAGE and western blotting

After collection, sperm were capacitated for 60 min in full capacitation m-TYH media (3.0 × 10^6^ sperm per treatment). After capacitation, sperm were centrifuged at 12,100 × g for 2 min, washed in 600 μl of cold PBS, centrifuged at 12,100 × g for 3 min and re-suspended in SDS-sample buffer (Laemmli, 1970). Samples were supplemented with 5% β-mercaptoethanol and boiled for 4 min before loading into 8% SDS/PAGE gels. Proteins were then transferred to PVDF membranes, and blockage of nonspecific binding and incubation with primary antibodies were done as follows. For OGT and O-GlcNAc, PVDF membranes were incubated in 5% bovine serum albumin (BSA, Sigma cat # A7906, St. Louis, MO) in Tris-buffered saline with 0.01% Tween-20 (TBS-T) for 1 h at room temperature; Western blotting was carried out using the following dilution of O-GlcNAc monoclonal antibody (clone CTD110.6) 1:2,000 (Cell Signaling, cat # 9875, Danvers, MA), O-GlcNAc monoclonal antibody (clone RL2) 1:1,000 (ThermoFisher, cat # MA1-072, Rockford, IL), and OGT polyclonal antibody 1:1,000 (Cell Signaling, cat # 5368, Danvers, MA) in 1% BSA/TBS-T overnight at 4°C with slow rocking. For phosphorylated PKA substrates, PVDF membranes were blocked with 5% milk in TBS-T for 1 h at room temperature; Western blotting was carried out by using a dilution of anti-pPKA monoclonal antibody 1:10,000 (Cell Signaling, cat # 9624, Danvers, MA) in 1% BSA/TBS-T overnight at 4°C with slow rocking. For Tyrosine phosphorylated proteins, PVDF membranes were blocked with 20% fish gelatin (Sigma cat # G7765, St. Louis, MO) for 1 h at room temperature; Western blotting was carried out by using a dilution of anti-pY antibody 1:10,000 (Millipore, cat # 05-321, Burlington, MA) overnight at 4°C with slow rocking.

In all cases, after three washes with TBS-T, incubation with the corresponding HRP-conjugated anti-mouse or anti-rabbit secondary antibody (1:10,000, Jackson Immunoresearch Laboratories, West Grover, PA) in TBS-T was conducted for 1 h at room temperature. Equal protein loading was determined by stripping membranes and re-probing with anti-tubulin antibody (1:5,000, clone E7, Hybridoma Bank, University of Iowa). Membranes were developed by using an enhanced chemiluminescence detection kit (ECLprime, GE Healthcare, Pittsburg, PA).

### Immunofluorescence

For mouse testis and epididymal tissue, samples were dehydrated, sectioned and rehydrated as described above for *in-situ* hybridization. Prior to staining, sections were treated with 1 % BSA in PBS-T blocking solution at 4°C overnight, followed by incubation with either O-GlcNAc clone CTD110.6 (1:100) or OGT antibody (1:100) in 1% BSA in T-PBS for 3 h in humidifier chamber at room temperature. Sections were then washed with PBS-T and incubated with the corresponding Alexa Fluo555-conjugated secondary antibody (1:1,000) diluted in 1% BSA in PBS-T for 3 h at room temperature; these solutions also contained Hoechst 33258 (1 μg/μl) for nuclear staining. After that, sections were washed three times for 5 min with PBS-T, and mounted using VectaShield (H-1000, Vector Laboratories, Burlingame, CA). Images were taken using a 20 × objective (Nikon, Plan Apo, NA 0.8) in a Nikon confocal Microscope.

For immunolocalization studies in epididymal sperm, samples were obtained as described above, and fixed in 4% paraformaldehyde (EMS, Hatfield, MA) in PBS for 10 min at room temperature. Sperm samples were centrifuged at 800 × g for 5 min, washed with PBS, and air-dried on Poly-L-Lysine-coated glass slides. Sperm were then permeabilized with 0.5% Triton X-100 in PBS for 5 min at room temperature and washed three times for 5 min with PBS and blocked with 10% BSA in PBS-T for 1 h at room temperature. After blocking, sperm were incubated with either O-GlcNAc antibody clone CTD110.6 (1:100), O-GlcNAc antibody clone RL2 (1:100) or OGT antibody (1:100) in 1% BSA in T-PBS over-night in humidifier chamber at 4°C. Following the incubation, sperm were washed with PBS-T and incubated with the corresponding Alexa Fluo555-conjugated secondary antibody (1:1,000) diluted in 1% BSA in PBS-T for 1 h at room temperature; these solutions also contained Alexa Fluo488-conjugated PNA (1:100) for staining of the mouse sperm acrosome and Hoechst 33258 (1 μg/μl) for nuclear staining. After that, samples were washed three times for 5 min with PBS-T and mounted as described above. Negative controls were run in parallel by incubation with secondary antibody alone. Images were taken using a 60 × objective (Nikon, PlanApo, NA 1.49) in a fluorescence microscope (Nikon Eclipse T300). Differential interference contrast (DIC) images were taken in parallel and served as control for sperm morphology.

### Statistical analysis

The software Infostat 2011 (www.infostat.com.ar) was used for the statistical analyses. Homogeneity of variances and normality were tested. Optical densitometry data comply with the parametric test requirements, then comparison between groups was performed by analysis of variance (ANOVA). When the ANOVA tests were significantly different between groups (*P* < 0.05), multiple comparisons were performed by Tukey's test. Immunofluorescence patterns frequency data were evaluated by non-parametric Kruskal-Wallis analyses. *P*-values *P* < 0.01 or *P* < 0.05 were considered significant as indicated in the Figure legends.

## Author contributions

MG, PV and PM designed research. DT and BP performed research. DT and MG analyzed data. DT, PV and MG wrote the initial draft.

### Conflict of interest statement

The authors declare that the research was conducted in the absence of any commercial or financial relationships that could be construed as a potential conflict of interest.

## Supplementary material

Supplemental Figure 1 Protein analysis of post-translational modification using O-GlcNAc antibody clone RL2 in epididymal sperm. Protein extracts of sperm collected from caput or cauda epididymis were separated by SDS-PAGE. **(A)** Western blotting of O-GlcNAcylated proteins (antibody Anti-O-GlcNAc clone RL2) from unwashed collection of caput and cauda sperm. Membranes were stripped and re-probed with anti-tubulin antibody to evaluate equal loading. *N* = 3. **(B)** Localization of O-GlcNAcylated proteins (O-GlcNAc clone RL2, green) in sperm recovered from the caput region (middle panel), scale bar 50 μm. DIC image of the same field of view (left panel). O-GlcNAc clone RL2 inset (right panel), scale bar 10 μm. **(C)** Localization of O-GlcNAcylated proteins (O-GlcNAc clone RL2, green) in sperm recovered from the cauda region (middle panel), scale bar 50 μm. DIC image of the same field of view (left panel). O-GlcNAc clone RL2 inset (right panel), scale bar 10 μm. Arrowhead indicates sperm head.
