# Supplementary figures and images for: Changes in Protein O-GlcNAcylation During Mouse Epididymal Sperm Maturation
Source: Front Cell Dev Biol. 2018 Jun 11;6:60. doi: 10.3389/fcell.2018.00060 (PMC6004373; doi:10.3389/fcell.2018.00060)

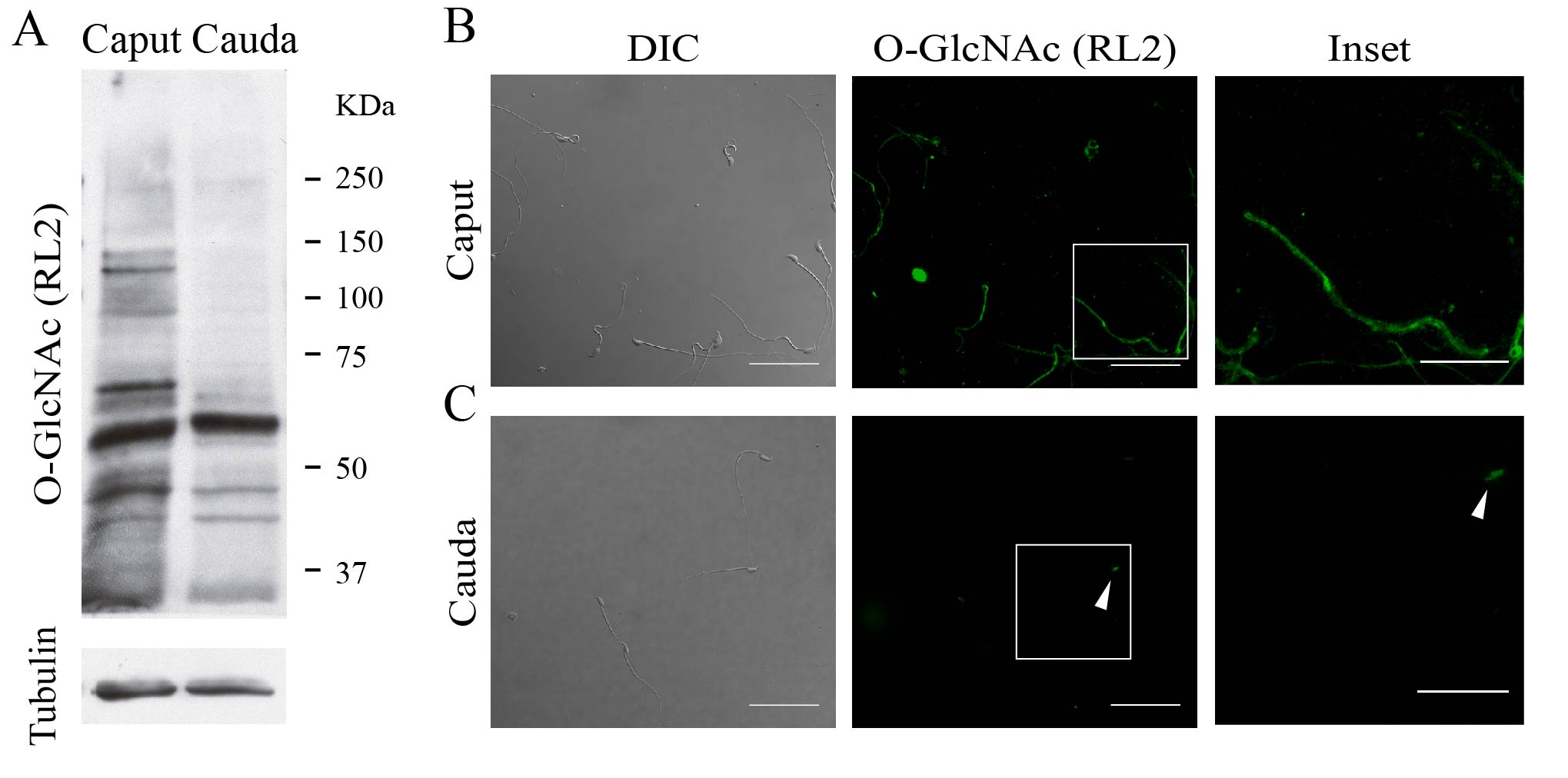

Supplement: Supplemental Figure 1 — Protein analysis of post-translational modification using O-GlcNAc antibody clone RL2 in epididymal sperm. Protein extracts of sperm collected from caput or cauda epididymis were separated by SDS-PAGE. (A) Western blotting of O-GlcNAcylated proteins (antibody Anti-O-GlcNAc clone RL2) from unwashed collection of caput and cauda sperm. Membranes were stripped and re-probed with anti-tubulin antibody to evaluate equal loading. N = 3. (B) Localization of O-GlcNAcylated proteins (O-GlcNAc clone RL2, green) in sperm recovered from the caput region (middle panel), scale bar 50 μm. DIC image of the same field of view (left panel). O-GlcNAc clone RL2 inset (right panel), scale bar 10 μm. (C) Localization of O-GlcNAcylated proteins (O-GlcNAc clone RL2, green) in sperm recovered from the cauda region (middle panel), scale bar 50 μm. DIC image of the same field of view (left panel). O-GlcNAc clone RL2 inset (right panel), scale bar 10 μm. Arrowhead indicates sperm head. [file Image_1.JPEG]
